# Clinicopathological spectrum of colorectal cancer among the population of the KwaZulu-Natal Province in South Africa

**DOI:** 10.11604/pamj.2020.37.74.21313

**Published:** 2020-09-18

**Authors:** Thandinkosi Madiba, Yoshan Moodley, Benn Sartorius, Kurt Sartorius, Colleen Aldous, Maseelan Naidoo, Vishendran Govindasamy, Shona Bhadree, Laura Stopforth, Yuming Ning, Pokala Ravi Kiran

**Affiliations:** 1 Gastrointestinal Cancer Research Group, Department of Surgery, University of KwaZulu-Natal, Durban, South Africa,; 2 Faculty of Health and Environmental Sciences, Central University of Technology, Bloemfontein, South Africa,; 3 London School of Hygiene and Tropical Medicine, London, UK,; 4 Department of Radiation and Oncology, University of KwaZulu-Natal, Durban, South Africa,; 5 Columbia University Medical Centre and Mailman School of Public Health, New York, USA

**Keywords:** Colorectal cancer, colorectal malignancy, colorectal carcinoma, rectal cancer, colonic cancer, epidemiology

## Abstract

**Introduction:**

the burden of colorectal carcinoma (CRC), once considered rare in Africa, may be changing with the disease being increasingly diagnosed and there is a suggestion that age and race influence tumour behaviour. We sought to describe the clinicopathological spectrum of CRC among the different race and age groups in a South African setting.

**Methods:**

analysis of prospectively collected data from an on-going colorectal cancer database, including demographics, clinical presentation, site, staging and grading on all patients enrolled over an 18-year period.

**Results:**

a total of 2232 patients with CRC were accrued over the study period (Africans, 798; Indians, 890; Coloureds, 104; and Whites, 440). Mean age was 57.7 (SD 14.4) but varied considerably by race (p < 0.001) with Africans being significantly younger. Young adults (aged < 40 years) totalled 305 and older patients (aged > 40 years) totalled 1927. The proportion of young patients (< 40 years old) was 28%, 7%, 9% and 3% among Africans, Indian, Coloured and White patients respectively. There were minimal variations in anatomical sub-site distribution. There was no difference in tumour stage between the various races and between older and young adults. Mucinous differentiation was more common in Africans and in young patients and poor differentiation was more common in African patients. Africans had a significantly lower resection rate compared to the other race groups (p < 0.001). Younger patients had a significantly lower resection rate compared to the older age group (p < 0.001).

**Conclusion:**

African patients were the youngest compared to the other race groups. Mucinous differentiation predominated in Africans and young adults. Poor differentiation predominated in Africans. Resection rate was lower for African patients and in young patients.

## Introduction

Colorectal cancer (CRC) is the third most commonly diagnosed malignancy and the fourth leading cause of cancer-related deaths worldwide, accounting for about 1.4 million new cases annually [1, 2] and almost 700,000 deaths in 2012 [1]. It occurs with great variability in the different parts of the world and it is not uniformly distributed in all race groups [1]. The distribution of CRC burden varies widely, with more than two-thirds of all cases and about 60% of all deaths occurring in countries with a high or very high human development index [1]. Although the number of cases of CRC in sub-Saharan Africa is thought to be very low in comparison to those diagnosed in high income countries (HICs), it constitutes a significant proportion of cancers in this region [3, 4]. In South Africa the incidence of CRC is increasing [5] and it has moved from being the 10^th^ most diagnosed cancer in the 1990´s [6] to its current status of being ranked among the foremost four cancers in both males and females [5, 7, 8]. There is suggestion in the literature that age [9, 10] and race [11] influence tumour aggressiveness, prognosis and disease stage. There is sparse literature on CRC in sub-Saharan Africa and there are even fewer epidemiological studies in this condition. We hypothesised that colorectal cancer occurs in all race groups and that there may be disparities in the clinicopathological spectrum of the disease. To address this hypothesis, we undertook an analysis of an on-going colorectal cancer database for the KwaZulu-Natal (KZN) Province of South Africa. We wanted to establish if the clinicopathological spectrum of colorectal cancer was homogenous in all race groups or would reveal certain differences. The manuscript was prepared according to the STROBE checklist.

## Methods

**Study setting:** the study was carried out at the Durban Colorectal Unit situated at Inkosi Albert Luthuli Central Hospital (IALCH), a tertiary referral hospital in Durban, South Africa. IALCH serves the eastern seaboard of the KwaZulu-Natal (KZN) Province of South Africa, which covers an area of over 92,000 km^2^. It houses the Colorectal and Oncology Units, both of which participate in the Gastrointestinal Cancer Multidisciplinary Team (MDT). Additional Colorectal and Oncology Units are situated at Addington Hospital (ADH) in Durban and Grey´s Hospital (GH) in Pietermaritzburg, both of which are subsidiary to the main units at IALCH. All patients with CRC cancer are discussed at the multidisciplinary clinics consisting of an MDT of surgeons, oncologists and radiologists. Members of the Colorectal Unit are also members of the MDT. The proposed treatment plan is thus collectively decided by the MDT.

**Study population:** the study included all patients with histologically proven colorectal cancer extracted from the ongoing colorectal cancer database which commenced in 2000 and is archived at the Department of Surgery, the University of KwaZulu-Natal (UKZN). Data of patients accrued from 2000 to 2017 were extracted for the purpose of this study. The enrolment into the database is dependent on the patients´ presentation at the UKZN-affiliated hospitals. The diagnostic approach depends on patient presentation. Patients with colonic cancers are generally managed surgically at the regional hospitals and are referred to the Multidisciplinary Clinic at IALCH only after resection, unless they present with complicated disease that requires management in a quaternary-level hospital. Patients with rectal cancers are referred to the Multidisciplinary Clinic before treatment for management decision. All the patient details, which include demographic details, stage of the disease, site of disease, years of survival, months of follow-up and treatment are captured. Race groups are defined as African, Indian, Coloured and White according to the criteria used by the South African Government [12]. In South Africa, “Coloured” refers to people of mixed ethnicity [12]. Patients were categorised into the four population groups and into young presenters (≤ 40 years) and older presenters (> 40 years).

**Colorectal cancer database:** the KZN colorectal cancer database is on-going and it commenced in 2000 and is archived in the Department of Surgery at UKZN. New patients are identified at the initial presentation at the Colorectal Units or at the time of arrival at the various oncology departments in the three hospitals with colorectal and oncology services. Follow-up data are collected from the colorectal and oncology records and entered onto the database.

**Study design and data collection:** this observational study is based on the on-going CRC database. The following key patient characteristics were captured namely, demographics, clinical presentation, tumour location and histopathological findings. Proximal colon was defined as the colonic segment from the caecum up to and including the transverse colon and the distal colon was the segment extending from the splenic flexure through descending colon and sigmoid colon [13-15]. Outcome measures were age at presentation, disease distribution, staging and tumour resectability.

**Data management and analysis:** the data were captured onto Microsoft Excel® and data analysis was conducted using SAS 9.4 (SAS Inc. Cary, NC). For the comparison of mean (SD), we used t-test for 2 groups or ANOVA (with Bonferroni correction) for > 2 groups. For the comparison of size (%), we used Chi-squared test. A p value of < 0.05 was considered statistically significant.

**Ethical considerations:** the study was approved by the Biomedical Research Ethics Committee of the University of KwaZulu-Natal (Ref No.: E198/04).

## Results

**Figure 1:**
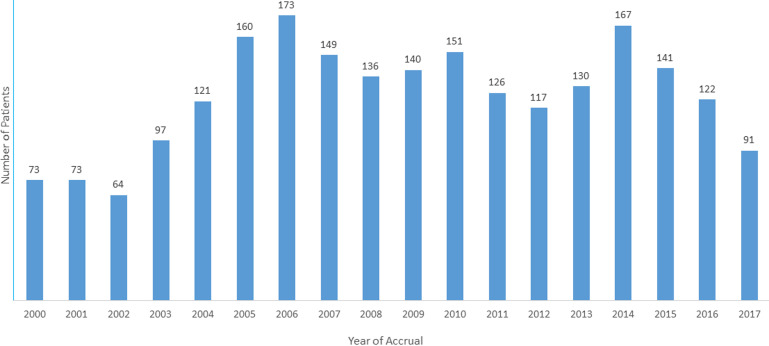
annual accrual of patients with colorectal cancer

**Figure 2:**
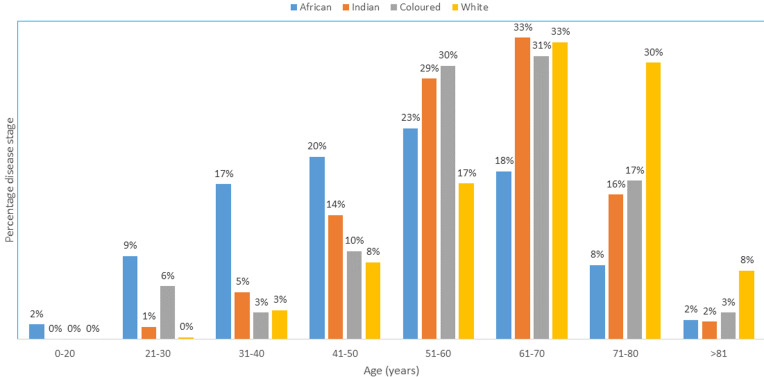
age at presentation stratified according to population group

A total of 2232 patients (1194 males) were diagnosed with CRC. The race groups included in the sample were African (798; 35.8%), Indian (890; 39.9%), Coloured (104; 4.7%) and White (440; 19.7%). Figure 1 shows the number of cases by year. Table 1 shows the findings stratified according to race groups. The mean age at presentation was lowest among Africans (p < 0.001). Of the 305 patients (13.67%) presenting at ≤ 40 years of age, Africans represented the majority (27.85%) with Whites having the least percentage at 3.2% (p < 0.001). Based on pairwise comparisons of age by race: Coloureds where 9.8 years older on average than Africans (p < 0.001); Indians were 9.6 years older on average than Africans (p < 0.001). Whites were 16.0, 6.1 and 6.3 years older on average than Africans, Coloureds and Indians respectively. No significant difference in mean age was observed when comparing Indians and Coloureds (p = 0.998). Figure 2 shows patient age at presentation stratified by race group. The peak was in the 6^th^ decade among Africans compared to the 7^th^ decade in the other three groups.

**Table 1:** demographic details of 2232 patients with colorectal cancer

	African n=798	Indian n=890	Coloured n=104	White n=440
Mean age (years)^a^	50.9 ± 15.5	59.7 ± 11.7	59.3 ± 13.1	65.7 ± 11.91
Age range (years)	13 - 103	21 - 91	26 - 84	21 - 92
Male	392	500	49	253
Male: female	1:1	1.3:1	1:1.2	1.4:1
Age ≤40 years	222 (27.85%)	59 (6.6%)	9 (8.7%)	15 (3.2%)
**Site distribution^b^**				
Proximal colon	194 (24.3%)	140 (15.7%)	17 (16.4%)	107 (24.3%)
Distal colon	203 (25.8%)	231 (26%)	33 (32.0%)	120 (27.3%)
Proximal: distal colon	1:1	1:1.6	1:1.9	1:1.1
Whole colon	397 (49.8%)	371 (41.70%)	50 (48.1%)	227 (51.6%)
Rectum	386 (48.4%)	492 (55.3%)	50 (48.5%)	210 (47.7%)
Colon: rectum	1:1	1:1.3	1:1	1.1:1
**Differentiation**				
Moderate	477 (59.8%)	624 (70.1%)	69 (66.4%)	296 (67.1%)
Mucinous	56 (7%)	36 (4.1%)	4 (3.9%)	9 (2.1%)
Poor	35 (4.4%	26 (2.9%)	2 (1.9%)	21 (4.8%)
Well	10 (1.3%)	19 (2.1%)	0	7 (1.6%)
Undifferentiated	2 (0.3%	4 (0.5%)	1 (1%)	2 (0.5%)
Not stated	217 (27.2%)	181 (20.3%)	28 (26.9%)	102 (24.1%)
**Staging**				
Stage I	41 (5.1%)	78 (8.8%)	8 (7.7%)	44 (10%)
Stage II	167 (20.9%)	214 (24.1%)	23 (22.1%)	115 (26.1%)
Stage III	196 (24.6%)	246 (27.6%)	22 (21.2%)	103 (23.4%)
Stage IV	229 (28.7%)	222 (24.9%)	31 (29.8%)	116 (26.3%)
Not staged	165 (20.7%)	130 (14.6%)	20 (19.2%)	62 (14.1%)
**Resection^d^**				
Resection	490 (61.5%)	624 (70.2%	72 (69.2%	323 (73.4%)

a Age: difference in age among populations p<0.001; Pairwise comparisons in terms of age: African vs White: p<0.008; African Coloured: p<0.008; African vs Indian: p<0.008; other races: p=NS. ^b^ Anatomical site: patients with involvement of multiple sites were excluded from this analysis. Pairwise comparison: African vs Coloureds: p=0.012; African vs Indians p= 0.006; African vs White: p=0.775; Coloured vs Indian: p=0.215; Coloured vs White: p=0.010; Indian vs Whites: p=0.006. Colon: rectal disease similar in all groups; ^c^ Staging: overall p=0.037; Africans vs Indians: p=0.004; Africans vs Whites: p=0.080. ^d^ Resection: overall p <0.0001; population comparisons: African vs White: p<0.008; African Coloured: p<0.008; African vs Indian: p<0.008; other races: p=NS

Distal colon disease was more common than proximal disease but this was more pronounced among Indian and Coloured patients. Pairwise comparison by race demonstrated that African patients had a significantly higher proportion of proximal disease compared to Coloureds and Indians (p = 0.012 and 0.006 respectively) but not significantly different when compared to Whites (p = 0.775). No significant difference was observed when comparing Coloureds to Indians (p = 0.215), however, Coloureds had significantly higher proportion of distal colonic disease compared to Whites (p = 0.010) as did Indians when compared to Whites (p = 0.006). Rectal disease predominated among Indians. The ratio of colon to rectal disease was 1:1 among African and Coloured patients. Colonic disease was slightly predominant among Whites. Tumour-related complications occurred in 541 (24.25%) including Africans (219; 27.5%), Indians (175; 19.7%), Coloured (33; 31.7%) and Whites (114; 25.9%) (p = 0.068). The tumour-related complication rate occurred in 91 young (≤40) patients (29.8%) and 450 older patients (23.4%) (p = 0.182). Moderately differentiated CRC was the most common differentiation in all race categories. Mucinous differentiation occurred more commonly among Africans at 6% and least frequently among Whites at 1%.

**Figure 3:**
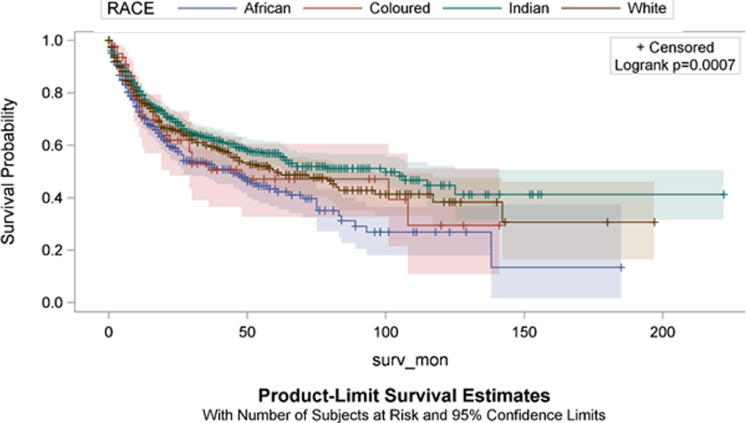
Kaplan-Meier curves of patient´s survival months stratified by race groups (surv-mon = survival in months)

**Figure 4:**
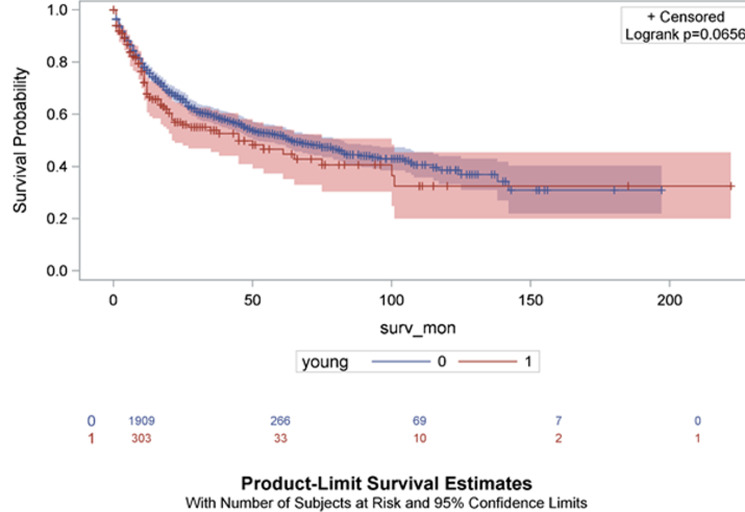
Kaplan-Meier curves of patient´s survival months stratified by youth of age groups (young = 0 if age > 40 years and young = 1 if age < 40 years); (surv-mon = survival in months)

African patients had the lowest resection rate at 61.5%. Pairwise comparison by race showed that Africans had a significantly lower resection rate compared to Coloureds, Indians or Whites (p = 0.025, p < 0.001 and p < 0.001 respectively). No significant differences in resection rates was observed when comparing Coloured to Indians, Coloureds to Whites and Indians versus Whites (p = 0.824, p = 0.633 and p = 0.619 respectively). Table 2 stratifies our findings by age group. Male to female ratio was similar in both age groups. Colonic disease distribution was similar in both groups and so was the colon: rectal disease ratio. There was no difference in staging. Mucinous differentiation was noted in 8% among young presenters and 4% among older presenters (p < 0.001). Poor differentiation was more common in younger patients (7% vs 3%) (p < 0.001). One hundred and ninety-nine out of 305 young patients underwent resection (65.3%) and 1311 out of 1927 older patients (68.1%) underwent resection (p = 0.3). Figure 3 shows the Kaplan-Meier curves of patients´ survival in months stratified by race groups. African patients had the lowest survival rate compared to the other race groups (p = 0.0007). Figure 4 shows the Kaplan-Meier curves of patient´s survival months stratified by youth of age groups (young = 0 if age > 40 years and young = 1 if age ≤ 40 years). Younger patients had a lower survival rate but this difference in survival did not reach statistical significance (p = 0.0656).

**Table 2:** demographic details of young and old patients with colorectal cancer

	≤40 years n=305	>40 years n=1927
Mean age (years)	32.2 ± 5.9	61.7 ± 10.7
Age range (years)	13-40	41-103
Male	161	1032
Male: female	1.1:1	1.2:1
**Site ^a^**		
Proximal colon	67 (22.0%)	391 (20.3%)
Distal colon	74 (24.3%)	515 (26.7%)
Colon	141 (46.2%)	905 (46.9%)
Rectum	156 (51.2%)	984 (51.1%)
**Stage ^b^**		
Stage I	24 (7.9%)	147 (7.6%)
Stage II	67 (22%)	451 (23.5%)
Stage III	77 (25.3%)	490 (25.4%)
Stage IV	82 (26.9%)	516 (26.8%)
Not stated	55 (18%)	322 (16.7%)
**Differentiation**		
Moderate	163 (53.4%)	1302 (67.6%)
Mucinous	27 (8.9%)	78 (4%)
Poor	22 (7.2%)	62 (3.2%)
Well	6 (1.9%)	30 (15.6%)
Undifferentiated	3 (1%)	6 (0.3%)
Not stated	84 (27.5%)	448 (23.3%)
**Resection ^c^**		
Underwent resection	199 (65.5%)	1311 (68.1%)

^a^ Patients with involvement of multiple sites were excluded from this analysis; proximal vs distal colon p=0.163; colon vs rectum p-0.353. ^b^ <40 years vs >40 years; p=0.916. ^c^ Resection p=0.3

## Discussion

This study has made a number of observations. Colorectal cancer was observed in all four race groups in the KZN Province. The South African population is 57,730,000 with KwaZulu-Natal Province comprising 20% of the population of whom 85%, 8.5%, 5% and 1.5% are African, Indian, White and Coloured respectively [16]. The large absolute number of African patients is not surprising because Africans constitute the majority of the population. However, the fact that Africans comprised a third of the patient cohort suggests that the incidence of CRC is less in Africans compared to the other race groups. Indians and Whites, who comprise 8.5% and 5% of the population respectively, constituted 40% and 20% of patients with CRC respectively. This greater relative proportion of affected Indian patients and White patients relative to their demographic representation in the whole province is possibly indicative of a larger contribution to the disease burden relative to the other race groups. African and White patients comprised the two extremes of age at presentation, with Whites mimicking the world literature with an age at presentation 66-70 years [6, 17] and Africans presenting a decade earlier, mimicking observations from the rest of the African continent which report age of presentation at a young age of 35-54 years [6, 18-24]. Although incidence rates vary slightly by ethnicity in the world literature, with higher rates among black and minority populations in HICs countries, male to female ratios remain largely consistent across different ethnic groups [4, 25]. The proportion of patients presenting at age ≤ 40 years in the world literature is reported at 1-5% [5, 6, 26].

The 28% of African patients presenting at the age of 40 years or below is much higher than the world average but it falls within the 18-36% reported in other South African studies [5, 6] and the 14-54% in other series in the African continent [6, 18, 27]. The 3.1% among White patients is similar to the 1-5% seen in the world literature. Although it is tempting to attribute the high prevalence in the young African patient to the possibility of hereditary non-polyposis colorectal cancer, we could not show this in our study. Nieminen *et al*. [28] observed that few, if any, of the young patients fulfilled the Amsterdam criteria nor could it be explained by other known hereditary syndromes. Pearlman *et al*. on the other hand, have observed that up to 16% of patients with early-onset CRC have pathogenic cancer susceptibility gene mutations [29]. Proximal and distal colonic disease occurred with similar frequency in Africans and Whites and distal colonic disease predominated in Indians and Coloureds. These findings contrast the findings of Cronje *et al*. [5] that African patients have predominantly proximal tumours and studies from North America which show that African-American patients tend to have more proximal disease [30-33]. The rectum was the most common site overall, as seen in all international studies [27]. Rectal disease was similarly more common in old and young patients in this series, which does not confirm the assertion by other authors that the proportion of rectal disease is higher in younger than older patients [34, 35]. Moderate differentiation was the most common in all race groups. There was a higher mucinous differentiation among African patients. Both poor differentiation and mucinous differentiation were more common in the young in this series, an observation made by others [5, 9, 36]. We did not demonstrate any racial predominance in terms of other tumour differentiation. We are also cognisance of the fact that these parameters are not the only ones that would predict tumour behaviour.

Stage IV disease ranged from 25-30% which fell at the upper limit of the 20-25% reported worldwide [37, 38]. Stage IV was relatively more common in Africans and Indian patients compared to the other race groups. Various authors have observed numerous racial/ethnic disparities in the risks of advanced-stage cancer and mortality among patients with CRC. Chien *et al*. found a considerable variation in the risks of advanced stage cancer across various race groups [30]. Data from the Surveillance, Epidemiology and End Results (SEER) program of the National Cancer Institute (NCI) [30] and the American College of Gastroenterology (ACG) Committee on Minority Affairs and Cultural Diversity [33] found that African-Americans had increased odds of late-stage CRC at the time of diagnosis relative to Whites. There was nothing in this series to suggest any similarities between African patients and African-American patients in terms of staging and resection rate. We did not confirm the findings of others that African patients in Africa tend to present at an advanced stage of the disease [5, 22, 27]. This late presentation is generally blamed, in Africa at least, on variable factors such as inefficient health services, socioeconomic factors, educational factors and different beliefs [4, 31]. There is evidence to suggest that socioeconomic status, access to CRC treatments and screening services and cultural and lifestyle factors are likely to contribute to the differences in stage of disease at presentation [30]. Data also show that young patients present with more advanced tumour stage at initial diagnosis, poor tumour cell differentiation and mucin production characterized CRC in these young patients [36]. This may have implications on the planning for screening of colorectal cancer in these countries. Whereas some studies suggest that CRC tends to be more aggressive in male patients [39], this was not apparent in this group of South African patients.

It was apparent in this series that age and race influenced the resection rate. African patients had a lower resection rate. The resection rate in African studies continent-wide is 60-80% [40, 41]. The results further showed a lower resection rate for younger patients, an observation that has been made by others [36]. Whereas it may be tempting to postulate that tumour aggressiveness is the reason for the lower resection rates, the similarity in staging and tumour-related morbidity across all categories does not support the notion of differences in tumour biology. Survival curves in this series suggest that survival was lower for African patients compared to the other race groups and for young compared to older patients. There are wide international inequalities in survival from colorectal cancer, even between economically developed countries [42]. Most notably, studies have revealed no significant difference in overall survival or cancer-specific survival between the young and old patients with CRC [43]. As can be seen from the survival curves in this series, there was a high attrition rate; this observation can be explained by high prevalence of stage III and IV disease both of which tend to have a high death rate [44]. The disparities observed in this study suggest that it is important to take into account the heterogeneity of broad racial categories when evaluating risks in these populations. Numerous differences have been observed in the risks of advanced-stage CRC and mortality across individuals in different population subgroups [11, 30, 45]. Contributing factors to racial differences observed in this series and other series include socioeconomic status, access to CRC treatments and screening services and cultural and lifestyle factors [30]. These observations suggest that it is important to take into account the heterogeneity of broad racial/ethnic categories when evaluating risks in these populations.

The study does have some limitations. The database reflects a single academic institution and the three affiliated tertiary hospitals. The database comprises patients diagnosed in the state hospitals and the 16-18% of the population treated in the private sector in South Africa are not captured in state archives [46, 47]. It is possible that some patients might not have been KZN residents, but rather residents of the surrounding provinces that border KZN Province. Similarly, patients living near the border might seek healthcare outside of the KZN Province and may not have been counted among demographic estimates. Furthermore, some patients with a diagnosis of CRC may have refused hospital admission and therefore not referred to the three main tertiary hospitals. The possibility of under-diagnosis does therefore remain a possibility. Some pathology reports did not comment on differentiation which may have led to under-reporting of these parameters. Furthermore, pathologic reporting was inconsistent with lymphovascular and perineural invasion not commented in the vast majority of patients and these parameters have been excluded from analysis. These findings relate to patients in the KZN region and should not be generalised to the rest of South Africa. Despite the limitations in terms of generalisability, evidence suggests that the trends seen in this study prevail in the rest of South Africa [5, 6, 24]. The strength of the study is that our database has the largest number of patients with CRC in an African country.

## Conclusion

CRC in our practice has a variable clinicopathological spectrum, which is population-based with Africans tending to present at a younger age and with a large proportion of young patients. Disease distribution, did not appear to show marked differences in the various race groups. Mucinous differentiation was more common among African patients, a finding that requires a more in-depth assessment. We are in agreement with Chien *et al*. [30] that developing screening and treatment programmes that target population groups at particularly high risks of poor outcomes of colorectal cancer may be an important means of reducing the disparities that we have observed in this series and those observed by others. Furthermore, the disparities observed in this study highlight the need for further in-depth assessment by studying the degree of local invasiveness and treatment-seeking behaviour patterns as well as molecular genetics and tumour biology of colorectal cancer, all of which are subjects of on-going research in our unit.

### What is known about this topic

- Colorectal cancer (CRC) is the third most commonly diagnosed malignancy and the fourth leading cause of cancer death in the world;
- The number of cases of CRC in sub-Saharan Africa is thought to be very low in comparison to those diagnosed in the high income countries;
- In South Africa, the incidence of CRC is increasing and it has moved from being the 10^th^ most diagnosed cancer to its current status of being ranked among the foremost four cancers in both males and females.

### What this study adds

- This study, with its large sample size, describes colorectal cancer in the different race groups in a South African setting;
- African patients tend to be younger compared to the other race groups; mucinous differentiation and poor differentiation predominates in Africans and mucinous differentiation predominates in young adults;
- Resection rate is lower for African patients and in young patients.
